# Bio-production of lactic and lactobionic acids using whey from the production of cow’s milk *Wagashi* cheese in Benin

**DOI:** 10.3389/fnut.2022.1020934

**Published:** 2022-10-17

**Authors:** Oumarou Djobo, Haziz Sina, Souriatou Tagba, Virgile Ahyi, Aly Savadogo, Adolphe Adjanohoun, Manuel Rendueles, Lamine Baba-Moussa

**Affiliations:** ^1^Laboratory of Biology and Molecular Typing in Microbiology, Department of Biochemistry and Cell Biology, University of Abomey-Calavi, Abomey-Calavi, Benin; ^2^Department of Chemical Engineering, IRGIB-Africa, Cotonou, Benin; ^3^Laboratory of Applied Biochemistry and Immunology, Department of Biochemistry and Microbiology, University Joseph KI-ZERBO, Ouagadougou, Burkina Faso; ^4^Benin National Institute of Agricultural Research, Cotonou, Benin; ^5^Department of Chemical and Environmental Engineering, University of Oviedo, Oviedo, Spain

**Keywords:** traditional cheese whey, lactobionic acid, lactic acid, *Pseudomonas taetrolens*, *Lactobacillus casei*

## Abstract

Traditional cheese is the main milk derivative in Bénin. This traditional process is not efficient and generate a lot of whey which has no real use until now. It is just disposed without being environmentally treated. Its use as a source for lactobionic and lactic acids production by *Pseudomonas taetrolens* and *Lactobacillus casei* is studied in this work, being also a proposal that can greatly boost economically the dairy sector in the country and reduce the end-of-cycle impact of the residue. To our knowledge, no data is available in the metabolization of Bénin’s traditional cheese whey and its potential transformation into commercially valuable products such as lactobionic and lactic acids. With bulk filtration, non-controlled pH batch fermentations and without nutrients supplementation, 66 and 22% of lactose in the traditional cheese whey have been converted into lactobionic acid and lactic acid using *Pseudomonas taetrolens* and *Lactobacillus casei*, respectively. Those are important results that encourage to enhance the bioprocesses used in a cost-effective way in order to scale up an industrial production.

## Introduction

In Benin, milk occupies an important part of dietary habits. As a highly perishable product, producers and retailers of fresh milk mainly turn to its transformation into cheese for its preservation (1). However, whatever the process used, the manufacture of cheese from milk gives an estimated yield of around 10% (kg/kg of milk) with variations depending on several parameters (2, 3). Despite efforts to optimize the yield of the traditional cheese-making process in Benin, the yield still remains low (4, 5). The production of Fulani cheese therefore generates a lot of whey. Whey has long posed enormous problems for the food industry, in particular, because of the need for its high chemical (COD) and biochemical (BOD) oxygen demand (6–8), prohibiting its dumping in waterways and even in municipal sewers (9, 10). From then, the use of whey as a carbon source for the bioconversion of its lactose into valuable products has been actively explored (10, 11). Specially, lactose has been successfully converted from whey into lactobionic acid and lactic acid using enzymes, chemically or by microorganisms (12–15). Lactobionic acid (LBA) and lactic acid (LA) find applications in a wide variety of fields such as food, pharmaceuticals, cosmetics, etc. (16). Lactobionic acid, because of its use in functionalization of biomaterials and targeted drug delivery (13, 17), has become one of the most promising specialty organics. Currently in Benin, whey does not find any application allowing its valorization on a large scale. The aim of this work is to study the bioconversion of whey from traditional cheese-making into lactic and lactobionic acids. Indeed, this way, we hope to set up a process of economic valuation of whey and boost the milk sector in our country.

## Materials and methods

### Microorganisms

Strains of *Lactobacillus casei* CECT 475 and *Pseudomonas taetrolens* LMG 2336 were kindly provided in freeze-dried form by the University of Oviedo, Department of Chemical Engineering and Environment. Lyophilisates were reactivated in nutrient broth (for *P. taetrolens*) and De Man, Rogosa and Sharpe (MRS) medium (for *L. casei*).

The reactivation phase of the freeze-dried *Lactobacillus casei* CECT 475 strains was carried out after two successive subcultures at 37°C for 2 h on liquid MRS medium (18).

The reactivation phase of freeze-dried *Pseudomonas taetrolens* LMG 2336 strains was carried out after two successive subcultures at 30°C for 2 h in nutrient broth (19).

After reactivation, the *L. casei* strains were then cultured on MRS agar containing: 2 g/L of dipotassium hydrogen phosphate, 20 g/L of glucose, 0.2 g/L of magnesium sulfate heptahydrate, 0.05 g/L manganous sulfate tetrahydrate, 8 g/L meat extract, 10 g/L peptone, 5 g/L sodium acetate trihydrate, 2 g/L triammonium citrate, 4 g/L extract yeast (Biokar Diagnostic, France). *P. taetrolens* was grown on nutrient agar containing 1 g/L meat extract, 2 g/L yeast extract, 5 g/L peptone and 5 g/L NaCl).

### Preparation of inocula

Fresh colonies of *P. taetrolens* were sought in previous nutrient agar plates. The colonies were isolated in 100 mL NB medium and then incubated with stirring at 250 rpm for 24 h.

The lyophilizate was suspended in 1 mL of sterile MRS medium then incubated at 37°C under anaerobic conditions at a maximum of 50 rpm. Decimal dilutions with 0.7% w/V NaCl were made and incubated, then the growth of a single type of colony was checked by simple observation (blue-violet strains). Previous strains were transferred to a bottle containing 90 mL of sterile MRS medium, lightly filled to simulate micro-aerophilic conditions. The bottle was incubated at 37°C for 20 h.

These precultures were subsequently employed to inoculate whey for batch fermentations.

### Preparation of whey

The whey were obtained fresh from local cheese makers. It was raw filtered, pasteurized at 95°C for 30 min, and the pH adjusted to 6.5 (where necessary) with NaOH before utilization. The physicochemical and microbiological quality of milk whey has been determined previously (20). Whey obtained from local cheesemaker in Akassato (Abomey-Calavi) contained: dry matter 5.66 ± 0.004 g/100 g, *p*H 5.21 ± 0.014, titratable acidity 0.35 ± 0.01, proteins 5.94 ± 0.091 g/100 g, lactose 43.2 ± 0.071 g/L.

### Batch culture

Colonies from the *P. taetrolens* inoculum were used to inoculate a 250 mL bottle of medium containing 250 mL of nutrient broth and incubated at 30°C at a shaking speed of 250 rpm for 10 h. Actively growing cells from these precultures were inoculated into 100 mL of pasteurized whey and incubated at 30°C at a shaking speed of 200 rpm for 72 h for the bioproduction of lactobionic acid.

For lactic acid bioproduction, a colony of *L. casei* from MRS agar was used to inoculate a 50 mL Erlenmeyer flask of medium containing 50 mL of MRS broth and then incubated at 37°C without shaking for 16 h. 10 mL of this culture was used to inoculate 90 mL of filtered whey and incubated at 37°C at a shaking speed of 100 rpm for 72 h.

During both fermentations, previous OD600–cell biomass calibration curves were established during preculture stages. For this purpose, cell biomass was determined in wet basis by centrifugation at 11,000 g for 10 min. On subsequent fermentation samples, only OD600 were measured spectrophotometrically and converted to cell mass using the calibration curve. The initial cell biomass for fermentations was 0.059 g/L for *L. casei* and 0.272 g/L and for *P. taetrolens*.

During the fermentations, samples were taken aseptically to determine bacterial growth, the evolution of lactose consumption, pH and the production of lactobionic and lactic acids. Samples were centrifuged to remove cell mass and other water-insoluble substances prior to analysis.

### Study of fermentation

Cell growth, specific production rate and the kinetic relationship between growth and acids production were investigated.

The growth of microorganisms has been studied using simple exponential-type relationship:


$$\frac{d\,X}{d\,t}=\text{μ}X$$

where *X* is the cell biomass, μ the specific rate of cell growth. This equation can easily be integrated between time *t*_0_ = 0 and any time *t* (in any time interval) to yield:


$$\mu \,\left(t\right)=\frac{\ln\left(X\right)-\ln\left(X_{0}\right)}{\left(t-t_{0}\right)}$$

The specific acid production rate is given by:


$$q_{p}=\frac{1}{X}\,\frac{d\,P}{d\,t}$$

Where *P* represents the product under consideration and *X* the biomass.

The Luedeking–Piret (21) type equation was used to study the relationship between cell growth and acids production rates:


$$\frac{d\,P}{d\,t}=\alpha \,\frac{d\,X}{d\,t}+\text{β}X$$

Dividing the previous equation by the biomass *X*, one can correlate the specific acid production rate to the specific growth rate:


$$q_{p}=\alpha \mu +\beta$$

The parameters α and β can be easily determined by comparing the data with the ordinary least squares method. This makes it possible to determine whether the production of the acids is linked to the cell growth (α is greater than β), not growth associated (α is smaller than β) or of mixed type (α is of the same order of magnitude as β). If, on the other hand, *q*_*p*_ decreases with an increase in μ, then the production is independent of the growth.

### Statistical analysis

Microbial growth was measured by UV spectrophotometry at 600 nm after centrifugation of the samples. Lactobionic acid, lactic acid and lactose concentrations were determined by high performance liquid chromatography coupled with a refractive index detector as described by Pedruzzi et al. (22). Data were recorded and preprocessed in an Excel workbook. Means, standard deviations and confidence intervals were calculated using R version 4.1.1 (23). Graphs were plotted using Gnuplot software 5.4 patchlevel 4.

## Results

### Bioproduction of lactobionic acid

**FIGURE 1:**
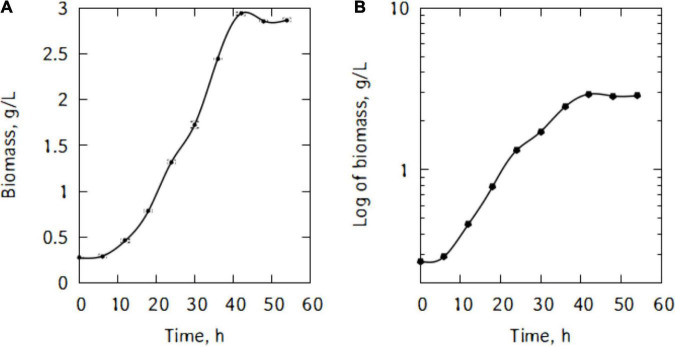
Growth of *P. taetrolens* in traditional cheese whey **(A)**, logscale **(B)**.

**FIGURE 2:**
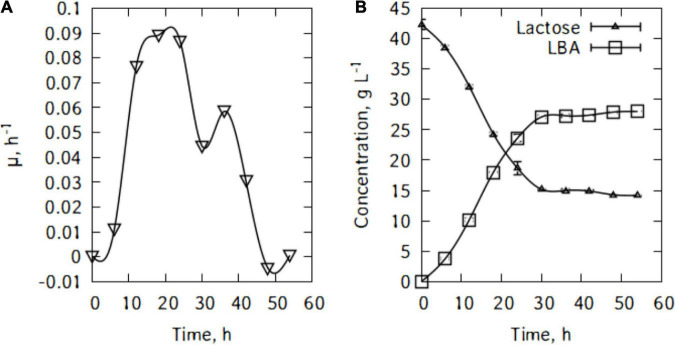
**(A)** Specific growth rate of *P. taetrolens* in traditional cheese whey, **(B)** lactose consumption and lactobionic acid production.

Figure 1A shows *P. taetrolens* growth in traditional cheese whey. From Figure 1B, it could be seen that the cells entered the exponential growth phase very quickly and extended to 42 h. This long exponential phase was followed by the stationary phase and eventually cells started declining from 48 h of fermentation. The maximum biomass was reached at the end of the stationary phase, after 42 h of fermentation (Figure 2). The maximum specific growth rate attained by the cells is 0.09 h^–1^. However, the specific growth curve had a non-smooth curve at the maximum, having a secondary maximum at 36 h (Figure 2A).

Figure 2B shows lactose consumption and lactobionic acid production by *P. taetrolens*. After the fermentation, the maximum LBA produced was 28.0 g/L and there were still 14.2 g/L of lactose left after 54 h of cultivation. *Pseudomonas taetrolens* cells were not able to metabolized all the lactose in the whey. As can be seen in Figure 2A, from 36 h of cultivation, the LBA output continued to increase but very slowly.

**FIGURE 3:**
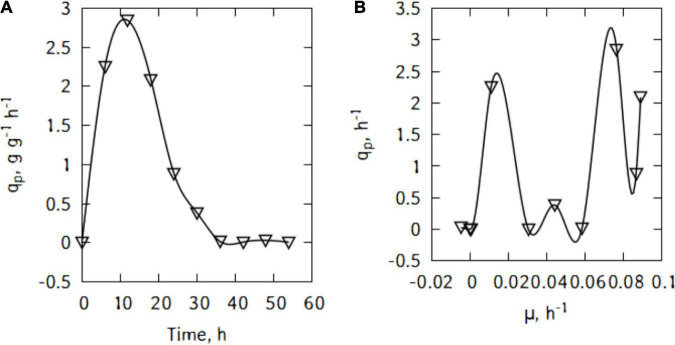
LBA productivity **(A)** and correlation between productivity and growth **(B)** of *P. taetrolens* in traditional cheese whey.

During batch cultivations, *P. taetrolens* displayed a maximum LBA productivity of 2.8 g/(g h) after just 18 h (Figure 3A). The productivity started decreasing quite slowly until 36 h of cultivation. Interestingly, Figure 3B suggests different types of relations between *q*_*p*_ and μ. Although, globally the productivity increased with increase in growth, no general pattern could be found and the curve alternate growth associated and growth independent patterns.

### Bioproduction of lactic acid

**FIGURE 4:**
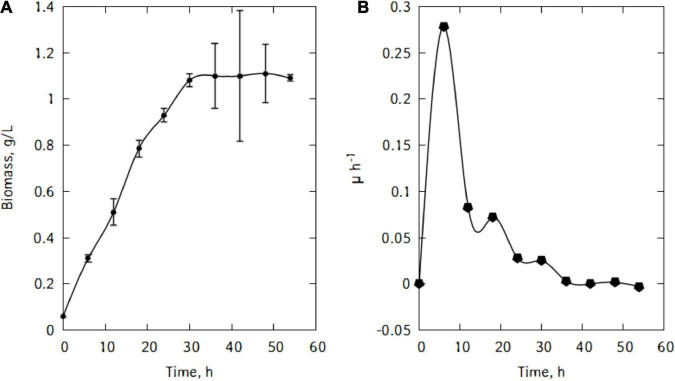
Growth of *L. casei* in traditional cheese whey **(A)**, specific growth rate **(B)**.

Figure 4 shows the evolution of the growth of *L. casei* cells in the traditional cheese whey. As could be seen in Figure 4A, the cells in the inoculum were already in the exponential growth phase. After inoculation, cells remained in this phase for 6 h. This rapid exponential phase was followed by a decrease and a stationary phase and eventually cells start declining. The maximum biomass was quickly reached at the end of the exponential phase (Figure 4B). The maximum specific growth rate attained by the cells were 0.28 h^–1^.

**FIGURE 5:**
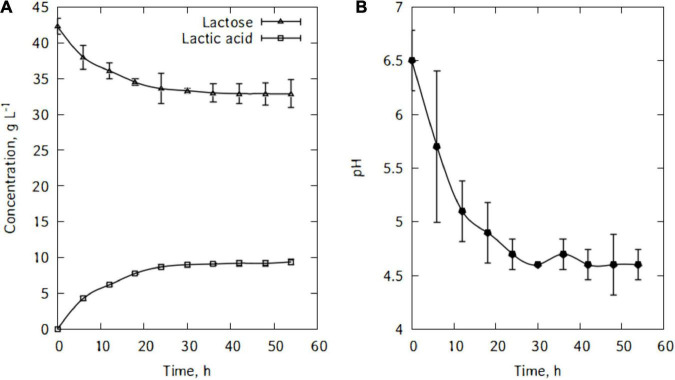
Evolution of lactose and lactic acid concentrations **(A)**, and pH during cultivation of *L. casei* in traditional cheese whey **(B)**.

Figure 5A shows lactose consumption and lactic acid production by *L. casei*. After the fermentation, the maximum lactic acid produced is 9.4 g/L and there were still 32.9 g/L of lactose left after 54 h of cultivation. *L. casei* cells were not able to metabolize major part of the traditional cheese whey’s lactose. As can be seen in Figure 5A, from 6 h of cultivation, the lactic acid output continued to increase but very slowly until the end of the fermentation. Figure 5B shows the evolution of pH during lactic acid production. The pH felt down quickly and reached a minimum of 4.6 within 30 h of fermentation.

**FIGURE 6:**
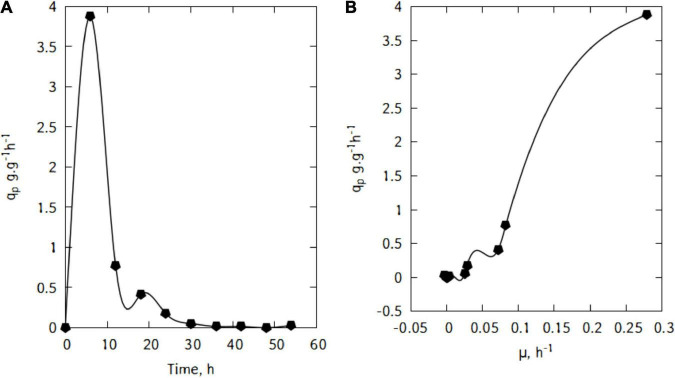
Specific lactic acid production **(A)**, and correlation between *q*_*p*_ and μ during cultivation of *L. casei* in traditional cheese whey **(B)**.

During this batch cultivations, *L. casei* displayed a maximum lactic acid specific productivity of 3.88 g/(g h) after just 6 h (Figure 6A). The productivity started decreasing quite quickly until 36 h of cultivation.

**FIGURE 7:**
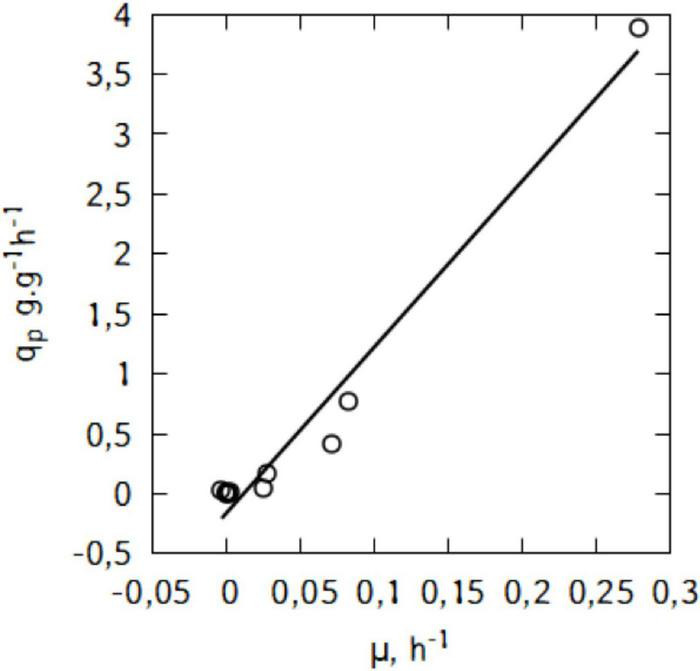
Relationship between specific lactic acid formation rate (*q*_*p*_) and specific growth rate (μ) during shake-flask cultivation of *L. casei* in traditional cheese whey.

Figure 6B suggests a linear relation between *q*_*p*_ and μ. This trend would suggest that the production of lactic acid in the conditions used here is growth-associated. Indeed, as shown in Figure 7, there is a strong relation (R^2^ = 0.97) and the Luedeking–Piret parameters are: α = 13.9 and β = -0.16 (α > > β).

## Discussion

The bioprocess used in this study aimed to investigate the usability of traditional cheese whey as substrate for production of high added value products such as lactobionic acid and lactic acid using now well-known bacteria. To serve as background for further investigations and real industrial production setup in Benin, the whey was used as is, and, only necessary bioprocess parameters have been controlled. Indeed, in both cases, no nutrients supplements were added for enhancing bacterial growth nor was the pH controlled.

Under the conditions of culture employed, the specific productivity achieved by *P. taetrolens* seed corresponds to a bioconversion yield of 66.43%. This yield is less than the 91% obtained by Alonso et al. (12). It is to noted, however, that these authors used different feed strategies, nitrogen supplementation, pH-shift conditions, purified whey and ultracentrifugation instead of pasteurization. However, the μ_*max*_ obtained here is similar to the one obtained by the same authors in 2017. The pattern observed between the specific LBA production rate and the specific growth rate has been used by the previous authors to decouple the LBA formation phase from *P. taetrolens*, a phenomenon that has been proved to make a good balance between cells metabolic status and resources utilization (24) especially in *Pseudomonads* (25). Indeed, Alonso et al. (14) showed that using a temperature-driven approach, the lactobionic acid formation can be completely decoupled from the growth of *P. taetrolens*. They also arrived to the conclusion that this approach could be used to boost the bioconversion ability of *P. taetrolens*, as typically, mixed-growth-associated production patterns result in low yields.

It is interesting that high LBA productivity was obtained here with a raw whey and basic process control. These results suggested that a complete metabolization of lactose in Benin’s traditional cheese whey would be possible. Indeed, selected cost-yield effective parameters control such as the temperature decoupling (12, 14, 26), the use of initial higher cell densities to accelerate the onset of the production (27) and whey concentration has been proven (13) to significantly improve the conversion of lactose to lactobionic acid by *P. taetrolens* cells.

This study is well suited for the investigation of the bioproduction of lactic acid, for, at industrial scale, it is produced using batch processes. The results obtained for the production of lactic acid are not to be compared to those of authors who used pure lactose often supplemented by oligo-elements and nitrogen sources like those of Büyükkileci and Harsa (28). A better yield is also obtained when whey permeate is used (29, 30) but those pre-processing operations and additions can be very expensive and could make the process economically unfavorable. From an industrial practical approach, the culture medium should be cost effective. Alonso et al. (31) obtained a lactic acid titer using *L. casei* on residual yogurt whey under non-controlled pH conditions higher than the titer obtained in this study under the same conditions. However, they supplemented their medium with yeast extract in the preculture stage. The low titer obtained was due principally to the rapid decrease in pH (after 12 h the pH reached around 4) which has a strong inhibitory effect on *L. casei*. On the other hand, Büyükkileci and Harsa (28) showed that while there was no substrate inhibition at concentrations up to 102 g lactose/L, lower lactose concentration yields lower lactic acid productivity.

In using lactic acid bacteria in general, temperature control is of major importance because its departure from what is required for optimum growth can substantially reduce the microbial activity and can subsequently lead to microorganisms’ complete inactivity (32).

In this lactic acid bioproduction experiment with *L. casei*, the pH dropped down quickly to 5.1 after 12 h of fermentation. According to a study by Büyükkileci and Harsa (28) using reconstituted lactose and *L. casei*, highest productivity value was obtained at pH 5.5. Although at other pH values similar trends were obtained, there was significant delay for complete lactose utilization (12 h at pH 5.5 and 23 h at pH 5.0). It can then be seen that after 12 h of fermentation, the lactose metabolism has begun to significantly decrease and reduce lactic acid production ability of the microorganism.

The respective results obtained here by *P. taetrolens* and *L. casei* for lactobionic and lactic bioproduction from traditional cheese whey (respectively) have important implications on the development of bioproduction systems of these two organic acids in Bénin. Indeed, strategies for increasing the processes yield can be easily setup such as those mentioned previously. However, overcoming the low-yield production is less tedious than establishing downstream processing strategies to purify the products, as this last step is the main challenge for any industrial bioproduction system implementation (33). Hence, before any industrial scaling be considered, the next steps to be investigated are the optimization of the yields and the cheapest way to purify the products.

## Conclusion

Traditional cheese whey in Benin, a waste material containing among other constituents, important concentrations of lactose, has been revealed as a suitable and direct substrate to lactobionic and lactic acids production by *P. taetrolens* and *L. casei*. Even with growth supplement not added and the pH not controlled, the process has shown a bioconversion efficient enough to be investigated further for industrial implementation. Supplementing with growth factors should increase the yields obtained. The evolution of the lactose content has shown how it is metabolized more efficiently by *P. taetrolens* than *L. casei*, in the actual culture conditions. Results suggests that *L. casei* was able to metabolize lactose within 24 h to produce 9.4 g/L lactic acid and *P. taetrolens* was able to metabolize most lactose within 36 h to produce 28.0 g/L lactobionic acid. In spite of the yields achieved, residual sugar content could be further reduced. Alternative fermentation strategies could be used to improve the efficiency compared with the traditional batch culture avoiding inhibition effects mainly for the lactic acid production. The results obtained are of great interest in the biotechnological use of traditional cheese whey in Benin, otherwise useless. This could be sustainable and interesting bioprocesses to obtain value-added industrial products from very cheap raw material.

## Data availability statement

The raw data supporting the conclusions of this article will be made available by the authors, without undue reservation.

## Author contributions

OD and MR conceived of the presented idea. OD, AA, HS, and ST conduced the trial set up and performed the experiments and computations. HS, LB-M, and AS verified the analytical methods. LB-M and VA encouraged OD to investigate and supervised the findings of this work. All authors discussed the results and contributed to the final manuscript.

## Abbreviations

LBA: Lactobionic acid
LA: Lactic acid
*q*_*p*_: Specific production rate
μ: Specific growth rate
α, β: Luedeking and Piret coefficients
*P*: Products (LBA or LA)
*X*: Biomass expressed as Dry Cell Weight or Optical Density
OD600: Optical Density at 600 nm.
